# RELATIONS BETWEEN ASTHMA AND OBESITY: AN ANALYSIS OF MULTIPLE FACTORS

**DOI:** 10.1590/1984-0462/2021/39/2019405

**Published:** 2020-11-06

**Authors:** Leticia Nabuco de Oliveira Madeira, Maria Alice Neves Bordallo, Marcos Antonio Borges, Agnaldo José Lopes, Isabel Rey Madeira, Fábio Chigres Kuschnir

**Affiliations:** a Universidade Estadual do Rio de Janeiro, Rio de Janeiro, RJ, Brazil.

**Keywords:** Asthma, Pediatric obesity, Child health, Interleukins, Spirometry, Asma, Obesidade pediátrica, Saúde da criança, Interleucinas, Espirometria

## Abstract

**Objective:**

Asthma and obesity are prevalent and interrelated diseases. In the pediatric population, the effect of systemic inflammation associated to obesity, leading to inflammation of the airways, is currently controversial. Our aim was to compare inflammatory, clinical and spirometric patterns between children with asthma and obesity and those within the normal weight status range.

**Methods:**

A total of 79 boys and girls from 6 to 10 years old were selected and divided into four groups: obese asthmatics, non-obese asthmatics, obese non-asthmatics, and non-obese non-asthmatics. In addition to collecting clinical and anthropometric data, all children underwent spirometry and skin prick tests for inhalant allergens. Blood samples for measurement of cytokines and adipokines were also collected.

**Results:**

Obese asthmatics had significantly worse control of asthma than non-obese asthmatics (OR 4.9; 95%CI 1.1‒22.1), regardless of sex, physical activity and atopy. No differences in spirometry, Th1 and Th2 cytokines and adipokines levels were observed among the four groups. The prick tests were positive in 81.8 and 80% of non-obese asthmatics and obese asthmatics, respectively.

**Conclusions:**

The degree of control of asthma was significantly lower in the obese group, regardless of the findings of no differences in spirometry. Extra-pulmonary factors could be responsible for this symptomatic profile. High positivity of skin test in both groups, which is considered a good marker of atopy, shows a preponderant atopic component in the genesis of asthma, both in children with obesity and in those within the normal weight status.

## RESUMO

**Objetivo:**

A asma e a obesidade são doenças prevalentes e inter-relacionadas. Na população pediátrica, o efeito da inflamação sistêmica associada à obesidade, levando à inflamação das vias aéreas, é controverso. Nosso objetivo foi comparar padrões inflamatórios, clínicos e espirométricos entre crianças obesas e aquelas com peso normal.

**Métodos:**

Setenta e nove meninos e meninas de 6‒10 anos de idade foram selecionados e divididos em quatro grupos: asmáticos obesos, asmáticos não obesos, não asmáticos obesos e não asmáticos não obesos. Além de dados clínicos e antropométricos, todas as crianças foram submetidas a espirometria e testes cutâneos para alérgenos inalantes. Também foram coletadas amostras de sangue para dosagem de citocinas e adipocinas.

**Resultados:**

Obesos asmáticos tiveram um controle significativamente pior da asma do que os não obesos (RP 4,9; IC95% 1,1‒22,1), independentemente do sexo, atividade física e atopia. Não foram observadas diferenças nos níveis de espirometria, citocinas Th1 e Th2 e adipocinas entre os quatro grupos. Os testes cutâneos foram positivos em 81,8 e 80% dos não obesos asmáticos e obesos asmáticos, respectivamente.

**Conclusões:**

O grau de controle da asma foi significativamente menor no grupo obeso, apesar de não ter havido diferenças nos achados espirométricos. Esse resultado sugere que fatores extrapulmonares podem ser responsáveis por esse perfil sintomático. A alta positividade do teste cutâneo nos dois grupos, considerado um bom marcador de atopia, demonstrou o componente atópico como preponderante na gênese da asma, tanto em crianças com obesidade quanto naquelas com peso normal.

## INTRODUCTION

Asthma and obesity are among the most prevalent diseases of childhood. Several studies show that the prevalence of asthma has increased in recent decades throughout the world.^1^ Obesity is considered by the World Health Organization (WHO) to be a worldwide epidemic, presenting an alarming growth over the last 20 years, especially in children.^2^ In this context, the association between these two prevalent diseases and their specificities is of great interest.

Longitudinal studies indicate that obesity precedes asthma and that the relative risk of asthma increases with obesity.^3^ A meta-analysis indicated a dose-response relation between body weight and the incidence of asthma, reporting a relative risk (RR) of asthma of 1.19 (95% confidence interval [95%CI] 1.03‒1.37) for overweight children and a RR of 2.02 (95%CI 1.16‒3.50) for children with obesity.^4^ In adults, weight reduction in obese asthmatics resulted in a decline in the severity and intensity of asthma symptoms.^5^

Obesity, like asthma, is considered a pro-inflammatory state. Systemic inflammation created by the excess adiposity is involved in the physiopathology of various conditions and has been identified as a possible mechanism for the development of asthma in individuals with obesity.^6^ Indeed, asthma and obesity appear to be connected in a multifactorial way. However, the intrinsic mechanisms and causality of this association have not been fully elucidated. Different hypotheses have been proposed to explain the pathogenesis of this association as changes in the mechanics of the airways, immune response, hormonal influences and genetic-environmental factors have been shown.^7^

In the pediatric population, however, the effect of systemic inflammation associated to obesity, leading to inflammation of the airways, is currently controversial. Thus, the objective of our study was to compare clinical, inflammatory and spirometric patterns of asthma in children with obesity and those within the normal weight status range to help clarify the interactions between these two entities.

## METHOD

A cross-sectional study with a population consisting of prepubescent boys and girls, from 6 to 10, was conducted between March 2016 and August 2018, at the Pediatric and Endocrinology clinics of Hospital Universitário Pedro Ernesto (Rio de Janeiro City, Brazil). All participants were previously consulted at least once in our hospital.

The study protocol was approved by the institutional Ethics and Research Committee, and all individuals responsible for the participants signed a consent form, which was written in accordance with the Declaration of Helsinki.

The research sample was of convenience, and patients were recruited according to the order of arrival for consultation. On this occasion, participants were allocated into four different groups: obese asthmatics, non-obese asthmatics, obese non-asthmatics and non-obese non-asthmatics. Patients on chronic use of oral corticosteroids or immunosuppressive treatments and chronic diseases other than asthma or obesity were excluded from the study.

Questionnaires containing items on demographic and socioeconomic data, asthma, rhinitis, use of medication, presence of passive smoking, family history of atopy and physical activity were completed. Clinical, anthropometric and immediate-reading skin tests for aeroallergens were then performed. Collection of laboratory tests and spirometry occurred within a maximum of one week.

The diagnosis of asthma was performed with the evaluation of a pediatric allergist, complemented by an affirmative answer to the following question: “Did your child present wheezing in the last 12 months?”, according to the asthma module of the International Study of Asthma and Allergies in Childhood (ISAAC), validated for Brazilian children.^8^

Asthma was classified according to the recommendations of the Global Initiative for Asthma 2012 (GINA) as follows:

- Controlled: daytime symptoms or rescue need ≤2x/week; absence of activity limitation and nocturnal symptoms or exacerbations; and peak of expiratory flow (PEF), or forced expiratory volume within 1 second (FEV1) of normal values.
- Partially controlled: presence of any of these manifestations: day time symptoms or rescue need ≥2x/week; any limitation of activities or night time symptoms or awakenings; and PEF or FEV1 <80% of predicted values.
- Not controlled: 3 or more manifestations of partially controlled asthma within the last 4 weeks.^9^

The clinical diagnosis of allergic rhinitis was also performed with a pediatric allergist evaluation, complemented by an affirmative answer to the following question: “In the last 12 months, did your child have a problem with sneezing or nasal discharge or nasal obstruction when not cooled or sealed?”, from the ISAAC rhinitis module, validated for Brazilian children.^10^

Family history of atopy was defined as the presence of a first degree relative with asthma, rhinitis or atopic dermatitis. Presence of atopy was determined by at least one positive immediate-reading skin test for aeroallergens.

Patients were classified according to physical activity as follows: 1) sedentary: walking or running less than 1 km/day and, when they were not in school, spent most of the time seated; or 2) active: walking or running at least 1 to 2 km/day, and when they were not in school, spent most of the time in active games.^11^

The weighing of children was carried out using a digital scale; height was measured using a wall stadiometer. Measurement of waist circumference was taken at the height of the iliac crests, following the recommendations by the National Health and Nutrition Examination Survey (NHANES) III.^12^ The anthropometric data were analyzed according to the weight, height and body mass index curves of the 2007 WHO guidelines. The following BMI criteria for age and sex were used to classify patients: eutrophic, between -2DP (standard deviation) and ≤+2DP; obesity:>+2DP, and severe obesity, >+3DP.^13^

To evaluate the waist circumference (WC), the reference standards used were from the National Center of Health and Survey (NCHS-2000), which considered normal measures to under the 90^th^ percentile for age and sex (which included data from individuals with mixed ethnicities).^12^

The following laboratory tests were performed: complete blood count (including eosinophil counts and neutrophils), blood glucose, total cholesterol and fractions, triglycerides, insulin, leptin, adiponectin, IL-4, IL-5, IL-6, IL-8 and TNF-α. Interleukins (IL-4, IL-5, IL-6, IL-8 and TNF-α) and adipokines (leptin and adiponectin) were measured using Luminex 200 equipment (Luminex Corporation, Austin, TX, USA). The methodology of simultaneous analysis of multiple analytes (multiplexing) was used. All tests were duplicated. The kits used for cytokines were Ultrasensitive Magnetic Custom Luminex kit (Life Technologies, Camarillo, CA, USA). Adipokines kits were from the manufacturer EMD Millipore (Darmstadt, Germany). The cytokines, adiponectin and leptin values were respectively described in pg/mL, μg/mL, and ng/mL.

In the evaluation of atopy, the following standardized allergen extracts (Immunotech^®^, Rio de Janeiro, Brazil) were used in skin tests of immediate hypersensitivity to aeroallergens: house dust mites (*Dermatophagoides pteronyssinus, Dermatophagoides farinae* and *Blomia tropicalis*), dog and cat epithelium and cockroaches (*Periplaneta americana* and *Blatella germanica*). Positive and negative controls used were histamine hydrochloride (1 mg/mL) and saline, respectively. The skin tests were performed by a single investigator, using the modified Pepys technique.^14^

Pulmonary functional evaluation was performed with simple spirometry and with the bronchodilator test. Measurements were obtained using HD CPL (nSpire Health, Inc., Longmont, CO, USA), in accordance with procedure rules and interpretation.^15^ Pulmonary function results were expressed as a percentage of predicted values for Brazilian children.^16^ All evaluations were performed by a single pulmonologist.

Variables were described through their frequency distributions, means and respective standard deviations, according to their characteristics. The comparison of averages of continuous variables between the two groups of asthmatics and between the four study groups were performed using Student’s t-test and analysis of variance using the Tukey test, respectively. The chi-square test, odds ratio (OR) and respective 95% confidence intervals (95% CI) were used to compare the distribution of categorical variables among the asthmatic groups. Logistic regression models were created to verify possible confounding factors. A “full” model was performed with all explanatory variables of clinical relevance. Correlations between spirometric variables, cytokines, Z scores of BMI (ZBMI) and waist circumference (WC), which showed a normal distribution according to the Kolmogrov-Sminorv test, were assessed using Pearson’s linear correlation. The same analyses were conducted separately for both sexes. The analyses were performed using SPSS statistical software for Windows, version 20.0 (IBM, Armonk, NY, USA). The significance level of 0.05 was considered for all tests performed.

## RESULTS

We studied 79 children, distributed among 4 study groups: (1) non-obese non-asthmatic; (2) non-obese asthmatic; (3) obese non-asthmatics and (4) obese asthmatics. Table 1 shows the distribution by sex and the means of age, ZBMI and WC in the study groups. There were no significant differences between the means of ZBMI (p=0.46) and WC (p=0.32) by sex.

**Table 1:** Socio-demographic and anthropometric characteristics of the studied population.

	Non-Obese Non-Asthmatic (n=16)	Obese Non-Asthmatic (n=21)	Non-Obese Asthmatics (n=22)	Obese Asthmatics (n=20)	p-value
Sex					
Female	7 (56.2%)	10 (47.6%)	5 (22.7%)	9 (45.0%)	0.66
Male	9 (43.8%)	11(52.4%)	17 (77.3%)	11 (55.0%)	
Age (years old)	8.4(1.5	8.9(1.4	7.9(1.7	8.4(1.6	0.19
Z score BMI	0.3(0.8	3.0(0.7	-0.1(0.8	2.9(0.6	<0.001
WC (cm)	59.9(5.6	85.3(15.0	56.6(6.1	79.0(12.7	<0.001

BMI: body mass index, WC: waist circumference

Table 2 compares clinical and lifestyle characteristics between the two groups of asthmatics. There were no significant differences regarding the presence of rhinitis, family history of atopy and skin tests of immediate reading between the non-obese asthmatic and obese asthmatics; positive skin tests were 81.8 and 80%, respectively.

**Table 2:** Comparison of clinical and socio-demographic characteristics between obese and non-obese asthmatics.

	Asthmatic Non-Obese (n=22)		Asthmatic Obese (n=20)		OR (95%CI)^a^	p-value	aOR (95%CI)^b^	p-value
	N	%	N	%				
Rhinitis	19	86.4	16	80.0	0.63 (0.12‒3.20)	0.44	0.44 (0.04‒4.40)	0.48
Family history of atopia	21	95.5	19	95.0	0.90 (0.05‒15.40)	0.73	5.30 (0.58‒47.80)	0.08
Positive skin test^c^	18	81.8	16	80.0	1.18 (0.23‒6.11)	0.58	3.30 (0.31‒36.00)	0.31
Use of corticosteroids^d^	8	36.3	5	25.0	0.65 (0.18‒2.34)	0.42	1.80 (0.20‒15.30)	0.25
Passive smoking	2	9.0	3	15.0	1.57 (0.23‒10.40)	0.22	3.80 (0.31‒37.30)	0.14
Physical activity								
-Active	12	70.6	12	60	0.62 (0.15‒2.40)	0.73	1.50 (0.20‒11.50)	0.67
-Sedentary	5	29.4	8	40				
Asthma Classification								
-Controlled	18	85.7	11	55	4.90 (1.08‒22.10)	0.043	18.30 (1.23‒271)	0.035
-Not Controlled	3	14.3	9	45				

^a^chi-square test; OR: Odds Ratio; 95%CI: Confidence Interval of 95%; ^b^logistic regression adjusted for sex and age; aOR: adjusted Odds Ratio; ^c^skin tests of immediate hypersensitivity to aeroallergens; ^d^beclometasone dipropionate: high dose (>200 mcg).

On the other hand, a comparison of the clinical severity criterion among the asthmatic groups revealed that, among non-obese patients, only 14.3% had partially controlled/uncontrolled asthma, whereas this percentage among obese patients was 45% (OR=4.99, 95%CI 1.08‒22.14, p=0.043). This association remained significant after adjusting for sex, age, and other categorical variables (Table 2).

Table 3 shows the main results of functional tests among asthmatics. No significant differences were observed in the distribution of spirometric mean values between the two groups. There was also no difference in the bronchodilator response with B2 agonist.

**Table 3:** Comparison of spirometric variables between obese and non-obese asthmatics.

	Asthmatic Non-Obese	Asthmatic Obese	p-value^a^
FVC (%)	92.8(13.1	89.1(11.8	0.34
FEV1 (%)	89.4(15.6	85.9(13.4	0.45
FEV1/FVC (%)	95.5(11.1	94.7(10.3	0.82
FEF2575(%)	94.4(33.1	82.1(24.2	0.18

^a^Student’s T-test. FVC: forced vital capacity; FEV 1: forced expiratory volume in 1 second; FEF2575: forced expiratory flow between 25‒75%

Table 4 shows the comparison between serum concentrations of cytokines, blood cellularity and adipokines in the four studied groups. No significant differences in the means of the evaluated cytokines were observed. The concentration of adiponectin was higher in the non-obese group than in the obese group, but this difference did not show any statistical significance. On the other hand, the mean values of leptin were significantly higher in the obese group than in other participants, regardless of the presence of asthma (p<0.001). These results remained significant when we analyzed boys (p<0.001) and girls (p=0.018) separately.

**Table 4:** Comparison of cytokines, adipokines and blood cellularity between the four study groups.

	Non-Obese Non-Asthmatic	Obese Non-Asthmatic	Non-Obese Asthmatics	Obese Asthmatics	p-value^a^
TNF- α	0.18(0.42	2.36(7.02	0.04(0.08	0.02(0.05	0.25
IL-6	0.30(0.39	1.58(1.97	1.00(2.00	4.26(9.28	0.12
IL-4	0.10(0.21	2.01(6.57	0.05(0.14	0.04(0.14	0.31
IL-5	0.20(0.45	0.28(0.51	0.47(0.75	0.82(1.53	0.32
IL-8	11.14(9.05	8.62(7.36	8.33(11.5	8.44(7.07	0.87
Adiponectin	49.9(33.3	30.2(41.6	63.1(46.4	32.5(23.6	0.07
Leptin	3.12(3.77	23.50(15.20	2.93(2.45	16.57(13.10	<0.001
Neutrophils	3236(966	3946(2526	4819(2634	5175(2432	0.38
Eosinophils	489(333	246(158	371(238	439(183	0.016

^a^Analysis of variance, Tukey's test. Expressed by means and standard deviation. TNF: tumor necrosis factor; IL: interleukin.

There were no significant correlations between spirometric variables and ZBMI and WC. Leptin was positively correlated with ZBMI (r=0.706, p<0.001) and with WC (r=0.804, p<0.001), whereas adiponectin was negatively correlated with ZBMI (r=-0.462, p=0.001) and WC (r=-0.361, p=0.013).

Significantly higher eosinophilia was observed in non-asthmatic non-obese patients compared to non-asthmatic obese subjects (p<0.01); however, there were no differences in this measure between the two asthmatic groups (p=0.31).

## DISCUSSION

The current medical literature shows that the association between asthma and obesity is still a subject of controversy, especially in children. Thus, we attempted to evaluate the relation between these diseases in a broader way, analyzing clinical, laboratory and spirometric aspects.

An important finding of our study was the significant difference in the clinical evaluation of disease control between the two groups of asthmatics, with the group of obese more symptomatic. On the other hand, there was no difference in the spirometric patterns and corticosteroid use between obese asthmatics and non-obese asthmatics. This finding suggests that extra-pulmonary factors could be responsible for the symptomatic profile. Sah et al. showed that, although obese individuals did not have worse asthma control, they presented more dyspnea, more nocturnal awakenings, lower quality of life associated to asthma and greater use of health services.^17^ It was postulated that this finding would be justified by changes in the chest wall (with worse respiratory dynamics) found in obese patients and not directly related to pulmonary pathology.^17^

Another study showed that, although reports of asthma, dyspnea and use of bronchodilators after exercise were more frequent in obese individuals, they had a lower possibility of airflow obstruction than in non-obese individuals, suggesting that a substantial number of obese individuals, who refer to themselves as asthmatics, are not truly asthmatics and are, thus, receiving inappropriate treatment.^18^ Similar results were found by Caprio et al., who showed that obese individuals with a self-reported asthma diagnosis, but without objective criteria in spirometric tests, had an increased perception of dyspnea during exercise, possibly associated to systemic inflammation and excessive ventilation for metabolic demands related to obesity.^19^

There was no difference in the level of physical activity between obese and non-obese children. These data are important to rule out a possible confounding bias, because there may be confusion between exercise intolerance related to asthma and poor physical conditioning.^20^

Our study did not show differences as to the presence of rhinitis, family history of atopy, eosinophilia, and neutrophilia between the two groups of asthmatics. These results, along with the high positivity of the skin test in both groups, which is considered a good marker of atopy, suggest a preponderant atopic component in the genesis of asthma, both in obese and non-obese children.

The increased levels of eosinophilia found in our study (>300 µl^-1^) in the asthmatic group are related in literature to greater severity of asthma,^21^ although such data should be analyzed with restrictions due to endemic parasitism in Brazil. This consideration is reinforced by increased levels of eosinophils in the group of non-obese and non-asthmatic children.

Recently, whether there are distinct inflammatory phenotypes that characterize obese asthmatics from non-obese asthmatics has been questioned. Unlike childhood “classical” atopic asthma, characterized by a Th2 phenotype with elevated levels of IL-4, IL-5 and IL-13,^22^ obesity-associated asthma would have a Th1 lymphocyte pattern, associated to elevated levels of TNF-α, IFN-γ and IL-6.^23^ Studies in obese asthmatic adults have shown an inflammation of the airways with a predominantly Th1 pattern, non-eosinophilic, and with significant neutrophilia.^24^ Results in children are more controversial. Visness et al. show an association between asthma and obesity, which was stronger in the absence of atopia.^25^ On the other hand, Yoo et al. reported that overweight children were more prone to be atopic when compared to non-obese children, suggesting that atopy could mediate the effect of adiposity on asthma.^26^

No differences in the inflammatory pattern of cytokines were demonstrated among study participants, regardless of the presence of obesity. Similarly, there were no differences in this distribution between the asthmatic groups, either in the cytokines associated to the Th2 response (IL-4, IL-5), or in those associated to the Th1 response (TNF-α, IL-6, IL- 8). There was also no significant correlation of cytokines with anthropometric measurements. Similar results were observed in a study by Rastogi et al., which showed no increase in airway or systemic inflammation through exhaled nitric oxide, sputum eosinophils, plasma C-reactive protein and IL-6 in children and adolescents aged 8 to 17 with asthma and obesity.^27^

Although there was no significant difference in adipokine concentrations between the two groups of asthmatics, the differences found in the serum levels of leptin and adiponectin among the obese children in relation to the non-obese ones reinforce the quality of the sample selection based on anthropometric measurements. Leptin is positively correlated with BMI and reflects body fat mass, unlike adiponectin.^7^

Our study has some limitations. The sample size may have limited the finding of differences among groups. In addition, the cross-sectional design hinders the inference of causality. Some other variables, such as the use of short-acting beta-agonists, could have been controlled, despite the low chance of interference in the final study result. However, due to the scarcity of studies in the studied age range, these results contribute to a better knowledge of these relations and could inform the direction of more comprehensive studies.

In conclusion, asthma in children does not appear to have a different inflammatory profile among those obese and non-obese, although obese asthmatics were significantly more symptomatic. Objective measures, such as pulmonary function tests, should be prioritized to avoid possible hyper-treatment of this group, considering that most exuberant symptomatology may not be related exclusively to asthma. The similar inflammatory profile suggests that the standard treatment with inhaled corticosteroids could be used in obese asthmatic children.

## References

[1] Solé D, Rosário NA, Sarinho ES, Camelo-Nunes IC, Barreto BA, Medeiros ML (2015). Prevalence of asthma and allergic diseases in adolescents:
nine-year follow-up study (2003-2012). J Pediatr (Rio J).

[2] United Nations Children's Fund, World Health Organization, World
Bank (2015). Levels and trends in child malnutrition: UNICEF-WHO-World Bank joint
child malnutrition estimates.

[3] Di Genova L, Penta L, Biscarini A, Di Cara G, Esposito S (2018). Children with obesity and asthma: which are the best options for
their management?. Nutrients.

[4] Chen YC, Dong GH, Lin KC, Lee YL (2013). Gender difference of childhood overweight and obesity in
predicting the risk of incident asthma: a systematic review and
meta-analysis. Obes Rev.

[5] Dixon AE, Holguin F (2019). Diet and metabolism in the evolution of asthma and
obesity. Clin Chest Med.

[6] Raucci R, Rusolo F, Sharma A, Colonna G, Castello G, Costantini S (2013). Functional and structural features of adipokine
family. Cytokine.

[7] Beuther DA (2009). Obesity and asthma. Clin Chest Med.

[8] Solé D, Vanna AT, Yamada E, Rizzo MC, Naspitz CK (1998). International Study of Asthma and Allergies in Childhood (ISAAC)
written questionnaire: validation of the asthma component among Brazilian
children. J Investig Allergol Clin Immunol.

[9] Global Initiative for Asthma (GINA) (2015). Global strategy for asthma management and prevention.

[10] Vanna AT, Yamada E, Arruda LK, Naspitz CK, Sole D (2001). International Study of Asthma and Allergies in Childhood:
validation of the rhinitis symptom questionnaire and prevalence of rhinitis
in school children in Sao Paulo, Brazil. Pediatr Allergy Immunol.

[11] Andersen RE, Crespo CJ, Bartlett SJ, Cheskin LJ, Pratt M (1998). Relationship of physical activity and television watching with
body weight and level of fatness among children. JAMA.

[12] Fernández JR, Redden DT, Pietrobelli A, Alisson DB (2004). Waist circumference percentiles in nationally representative
samples of African-American, European- American, and Mexican-American
children and adolescents. J Pediatr.

[13] de Onis M, Onyango AW, Borghi E, Siyam A, Nishida C, Siekmann J (2007). Development of a WHO growth reference for school-aged children
and adolescents. Bull World Health Organ.

[14] [No authors listed] (1993). Allergen skin testing. Board of Directors. American Academy of
Allergy and Immunology. J Allergy Clin Immunol.

[15] Miller MR, Hankinson J, Brusasco V, Burgos F, Casaburi R, Coates A (2005). Standardization of spirometry. Eur Respir J.

[16] Burity EF, Pereira CA, Rizzo JA, Brito MC, Sarinho ES (2013). Reference values for spirometry in school
children. J Pediatr (Rio J).

[17] Sah PK, Gerald Teague W, Demuth KA, Whitlock DR, Brown SD, Fitzpatrick AM (2013). Poor asthma control in obese children may be overestimated
because of enhanced perception of dyspnea. J Allergy Clin Immunol Pract.

[18] Sin DD, Jones RL, Man SF (2002). Obesity is a risk factor for dyspnea but not for airflow
obstruction. Arch Intern Med.

[19] Carpio C, Villasante C, Galera R, Romero D, Cos A, Hernanz A (2016). Systemic inflammation and higher perception of dyspnea mimicking
asthma in obese subjects. J Allergy Clin Immunol.

[20] Jago R, Salway RE, Ness AR, Shield JP, Ridd MJ, Henderson AJ (2019). Associations between physical activity and asthma, eczema and
obesity in children aged 12-16: an observational cohort
study. BMJ Open.

[21] Sánchez-García S, Mena AH, Quirce S (2017). Biomarkers in inflammometry pediatric asthma: utility in daily
clinical practice. Eur Clin Respir J.

[22] Gelfand EW, Joetham A, Wang M, Takeda K, Schedel M (2017). Spectrum of T-lymphocyte activities regulating allergic lung
inflammation. Immunol Rev.

[23] Rastogi D, Holguin F (2017). Metabolic dysregulation, systemic inflammation, and pediatric
obesity-related asthma. Ann Am Thorac Soc.

[24] Scott HA, Gibson PG, Garg ML, Wood LG (2011). Airway inflammation is augmented by obesity and fatty acids in
asthma. Eur Respir J.

[25] Visness CM, London SJ, Daniels JL, Kaufman JS, Yeatts KB, Siega-Riz AM (2010). Association of childhood obesity with atopic and nonatopic
asthma: results from the National Health and Nutrition Examination Survey
1999-2006. J Asthma.

[26] Yoo S, Kim HB, Lee SY, Kim BS, Kim JH, Yu JH (2011). Association between obesity and the prevalence of allergic
diseases, atopy, and bronchial hyperresponsiveness in Korean
adolescents. Int Arch Allergy Immunol.

[27] Rastogi D, Canfield SM, Andrade A, Isasi CR, Hall CB, Rubinstein A (2012). Obesity- associated asthma in children: a distinct
entity. Chest.

